# Benefit Effect of *Dendrobium officinale* Ultrafine Powder on DSS-Induced Ulcerative Colitis Rats by Improving Colon Mucosal Barrier

**DOI:** 10.1155/2021/9658638

**Published:** 2021-12-30

**Authors:** Xiang Zheng, Tao-Xiu Xiong, Ke Zhang, Fu-Chen Zhou, Hui-Ying Wang, Bo Li, Ying-Jie Dong, Xinglishang He, Lin-Zi Li, Qiao-Xian Yu, Gui-Yuan Lv, Su-Hong Chen

**Affiliations:** ^1^Zhejiang University of Technology, Hangzhou 310014, Zhejiang, China; ^2^Zhejiang Synergetic Traditional Chinese Medicine Research and Development Co., Ltd., Deqing 322099, Zhejiang, China; ^3^Zhejiang Senyu Co., Ltd., Yiwu 322099, Zhejiang, China; ^4^Zhejiang Chinese Medical University, Hangzhou 310053, Zhejiang, China

## Abstract

**Materials and Methods:**

After intragastric administration of DOFP for 3 weeks, the rat UC model was made by the administration of 4% oral DSS solution for one week, and the drug was given at the same time. During the experiment, the disease activity index (DAI) score of the rats was regularly computed. At the end of the experiment, the blood routine indexes of rats were obtained. The histopathological changes in the colon were monitored by hematoxylin-eosin (H&E) and PAS staining and observation of ultrastructural changes in the colon by transmission electron microscope. Occludin expression in the colon was monitored by Western blot, the expression of claudin-1 and ZO-1 in the colon was detected by immunofluorescence, and the expression of TNF-*α*, IL-6, and IL-1*β* in the colon was detected by immunohistochemistry.

**Results:**

The results firstly indicated that DOFP could significantly alleviate the signs and symptoms of the DSS-induced rats UC model, which manifested as improvement of body weight loss, increase of colon length, and improvement of the symptoms of diarrhea and hematochezia. Then, results from histopathology, blood routine examination, and transmission electron microscope analysis further implied that DOFP could dramatically reduce inflammatory cell infiltration and restore intestinal epithelial barrier integrity. In addition, the experiments of Western Blot analysis, immunofluorescence, and PAS staining also further confirmed that DOFP could markedly increase related protein expressions of the intestinal barrier and mucus barrier, as the expression of occludin, claudin-1, and ZO-1 in the colon significantly decreased. The experiments of immunohistochemistry confirmed that DOFP could markedly decrease protein expression levels of inflammatory cytokines TNF-*α*, IL-6, and IL-1*β*.

**Conclusion:**

DOFP notably alleviated inflammatory lesions, repaired the colon mucosa damage by promoting the expression of tight junction proteins occludin, claudin-1, and ZO-1 and inhibiting the release of inflammatory factors TNF-*α*, IL-6, and IL-1*β*, and finally achieved the purpose of treating UC.

## 1. Introduction

Ulcerative colitis (UC) is an inflammatory bowel disease characterized by inflammatory cell infiltration into the colonic mucosa. The etiology and pathogenesis of ulcerative colitis are not clear [1, 2]. This disorder severely affects the normal life of patients and also increases the risk of secondary infections and colon cancer [3]. At present, the drugs used to treat UC are corticosteroids, 5-aminosalicylic acid (5-ASA), immunosuppressants, and biological agents or their derivatives [4]. However, the side effects of these drugs have a negative impact upon the liver, as well as psychological functions and other body systems [5]. Therefore, there is an urgent need to obtain therapeutic drugs with definite curative effects and few side effects.

Although the exact pathophysiological mechanism of UC is not clear, studies suggest that diet, environment, and intestinal mucosal injury are closely related to the occurrence of UC [6–8]. Tight junctions (TJs) mainly include transmembrane proteins, occludins, claudins, cytoplasmic, and attachment proteins of the ZO family proteins. In-depth studies have found that the expression of TJ protein is abnormal in UC, and this directly affects the intestinal mucosal barrier function and plays a key role in promoting the occurrence and development of UC [9]. Intestinal epithelial TJ protein can not only block the abnormal immune response caused by pathogenic intestinal microorganisms but also alleviate the inflammatory response caused by excessive leakage of bacteria and antigens through the mucous membrane [8]. Therefore, when there is a decrease in TJs, intestinal permeability increases, and this can cause celiac disease, diabetes, UC, and other diseases [10].

*Dendrobium officinale* Kimura et Migo *(D. officinale*) is a plant that has the effect of “benefiting the stomach and promoting fluid” [11], and its uses were recorded in the Shennong Materia Medica over 2000 years ago. The dried stem is the plant part of *D. officinale* that is medicinally used, and modern pharmacological studies have shown that *D. officinale* has a variety of biological activities, such as hepatoprotective, anticancer, hypoglycemic, antifatigue, and gastric ulcer protection [12]. *D. officinale* has a variety of active principles, such as polysaccharides, flavonoids, alkaloids, and essential oils. And polysaccharides are the dominant substances [13]. It has been reported that *D. officinale* and its polysaccharides have many beneficial effects on UC, such as decreasing colonic inflammation and regulating intestinal flora [14–16]. Ultrafine grinding technology uses mechanical or hydrodynamic methods to crush raw materials into a nanopowder or micropowder. After ultrafine pulverization, the particle size of a drug can reach 1–10 *μ*m as an ultrafine powder [17]. The powder has the advantages of convenient transportation and storage, excellent water solubility, low loss of functional components, high utilization rate, and beneficial dietary fiber [18, 19].

Previous laboratory studies have shown that *D. officinale* ultrafine powder (DOFP) could increase the expression of intestinal ZO-1 in convalescent nonalcoholic fatty liver model mice and regulate the abnormality of the intestinal-liver axis by inhibiting LPS-TLR4-related inflammation [18]. However, whether *D. officinale* can inhibit intestinal inflammation by regulating TJ protein in the UC model requires further study. We speculated that DOFP might act on the TJ proteins of the intestinal mucosal barrier to subsequently ameliorate UC. Therefore, in this study, dextran sulfate sodium (DSS) was used to induce UC in rats, and the focus was on the TJ proteins occludin, claudin-l, and ZO-1 to explore the efficacy and mechanism of DOFP in the prevention and treatment of ulcerative colitis.

## 2. Materials and Methods

### 2.1. Materials and Reagents

DSS (relative molecular weight 40000, Hubei Xinghengkang Chemical Technology Co., Ltd.) and the reagents of hematoxylin and eosin were purchased from the Institute of Biological Engineering of Nanjing Jiancheng Co. Ltd. (Nanjing, China).

### 2.2. Animal Treatment

Forty healthy male SD rats (200 ± 20 *g*) were purchased from the Zhejiang Experimental Animal Center (Hangzhou, China), license number: SCXK (Zhejiang) 2014–0001. All the animals were housed at room temperature of 25°C and at 45–55% relative humidity with a 12-hour light-dark cycle. All rats were fed a standard pellet chow diet, and the gastric volume was 1 mL/100 g. All animal experiments were conducted with the approval of the Animal Care and Use Committee of Zhejiang University of Technology (ethical approval number: 20200603038).

### 2.3. DOFP Preparation

Dried *D. officinale* was purchased from Zhejiang Senyu Co., Ltd. (Zhejiang, China). The dried herb was crushed to create *D. officinale* coarse powder (DOFC) and *D. officinale* ultrafine powder (DOFP). The DOFP and DOFC were observed by scanning electron microscope (JEOL JSM-IT100, Beijing, China), the samples were fixed on scanning electron microscopy stubs using double-sided adhesive tape and then coated with Au at 50 mA for 3 min through a sputter coater, and a scanning electron microscope with a secondary electron detector was used to obtain digital images of the samples at an accelerating voltage of 20 kV.

The content of polysaccharides was determined by the phenol sulfuric acid method, and the maximum absorbance was found at 488 nm [18]. With standard glucose (Shanghai Yuanye Bio-Technology Co., Ltd., Shanghai, China) as the control substance, 1 mL extract sample or glucose standard solution was added to a 10 mL plugged test tube, and water was added to 1 mL, mixed with 1 mL 5% phenol (Shanghai Yuanye Bio-Technology Co., Ltd., Shanghai, China), then mixed with 5 mL sulfuric acid (Sinopharm Chemical Reagent Co., Ltd, Shanghai, China), reacted in a boiling water bath for 20 min, and cooled in an ice bath for 5 min to stop the reaction. The absorbance was measured at 488 nm. The standard regression equation for glucose is *y* = 0.0364*x*−0.0027; *R* = 0.9984 (*y* denotes absorbance, while *x* denotes the concentration of glucose).

### 2.4. Animal Groups

Rats were randomly divided into four groups, the normal group (NG), model group (MG), DOFP low-dose group (DOFP-L, at a dose of 0.3 g/kg), and DOFP high-dose group (DOFP-H, at a dose of 0.6 g/kg), with ten animals in each group. The DOFP-L and DOFP-H rats received the corresponding drugs on a daily basis (p.o.) for 4 weeks. After 3 weeks of treatment, except for the NG, 4% DSS solution was added to all other groups of rats drinking water ad libitum to induce ulcerative colitis (UC). For the 4 weeks experiment, all rats were fed with the standard diet, and the rats in the NG and MG were provided with water every day (p.o.). Referring to the Pharmacopoeia of the People's Republic of China (2020 Edition), the recommended daily dosage of *Dendrobium officinale* is 6–12 g. The administration dosage for the rats was converted according to the surface area of a 60 kg human body, the high-dose *Dendrobium officinale* ultrafine powder is 0.6 g/kg, and the low dose is 0.3 g/kg in this study. In addition, previous laboratory studies have shown that the 0.6 g/kg dose group of *Dendrobium officinale* ultrafine powder can increase the expression of tight junction protein in the intestine of nonalcoholic fatty liver model mice during the recovery period, inhibit LPS-TLR4 related inflammation, and regulate the abnormality of the intestinal-liver axis [18].

### 2.5. Disease Activity Score

After the establishment of the model, the body weight, bleeding, and fecal consistency of the rats were measured and recorded every day (Table 1) [20].

### 2.6. Blood Routine Index Detection

On the last day of the experiment, 12 hours after fasting, retroorbital blood was sampled and placed in an EP tube pretreated with Ethylene Diamine Tetraacetic Acid (EDTA) to prepare whole blood. The white blood corpuscles (WBC), red blood corpuscles (RBC), hemoglobin (HGB), hematocrit (HCT), mean cell volume (MCV), mean corpuscular hemoglobin (MCH), mean erythrocyte hemoglobin concentration (MCHC), platelet count (PLT), mean platelet volume (MPV), and eosinophil (EO) counts were analyzed using an automatic hematology analyzer (Sysmex Corporation, XT-2000i, Shanghai, China).

### 2.7. Colonic Length Measurement and Tissue Preservation

After anesthetization with pentobarbital sodium (Chengdu Huaxia Chemical Reagent Co., Ltd., Sichuan, China), blood samples were removed from the abdominal aorta of rats, then the colon was dissected and quickly separated, and the length of colon was measured. Part of the colon was stored at −80°C for Western blot examination; another part was cut into small pieces and fixed in 2.5% glutaraldehyde solution to be observed by transmission electron microscope; and the remainder was fixed in 4% neutral formalin for H&E staining, PAS staining, and immunofluorescence analysis.

### 2.8. Colon Tissue H&E Staining and Histopathological Score

The fixed colon tissue was embedded in paraffin after dehydration, and 4 *μ*m paraffin sections were prepared. After dewaxing and hydration, H&E staining was performed. The paraffin sections were observed and photographed by optical biological microscope and scored according to a standard procedure (Table 2) [21].

### 2.9. PAS Staining

For staining, 4 *μ*m paraffin sections were prepared. After dewaxing and hydration, the samples were treated with periodic acid dye, and incubation was performed for 6.5 min. After rinsing with distilled water, the samples were treated with Schiff reagent and were incubated away from light for 15 min and then rinsed with running water. Then, hematoxylin staining was performed for 1.5 min, and the samples were subsequently rinsed with running water and then dried. The tissues were observed, and images were recorded with an optical biological microscope.

### 2.10. Ultrastructural Observation of Colon in UC Rats

For structural examination, 2–3 pieces of colon tissue were submerged in 2.5% glutaraldehyde fixative solution for more than 24 hours. Then, ultrathin sections were created, and the ultrastructure of colonic epithelial cells was observed by transmission electron microscope (HITACHI, HT7700, 80 kV, China).

### 2.11. Western Blot Analysis

Liquid nitrogen was used for the mechanical lysis of 50–100 mg frozen colon tissues. Then, the tissues were lysed in RAPI buffer with protease/phosphatase inhibitor for 30 min on ice. The lysates were clarified by centrifugation at 10,000 rpm for 20 min at 4°C, and the protein concentrations were determined using BCA protein analysis kits (Shanghai Beyotime Biotechnology Co., Ltd., Shanghai, China). After adjusting the concentration, electrophoretic separation and membrane transfer were carried out. The membrane was incubated in 5% milk at room temperature for 2 hours, and the first antibody of occludin or GAPDH (Proteintech Group, Shanghai, China) was incubated with the membrane overnight at 4°C. After three washes in TBST for 5 min each, the second antibody was incubated with the membrane at room temperature for 2 hours. After rinsing with TBST, the bands were identified by chemiluminescence, and the gray value of the corresponding protein band was analyzed by ImageJ software.

### 2.12. Immunofluorescence

For immunofluorescence staining, 4 *μ*m paraffin sections were prepared, and after dewaxing and hydration, the samples were incubated in 3% H_2_O_2_ for 15 min at room temperature. After three washes in PBS for 5 min each, the samples were incubated in 5% BSA at room temperature for 2 hours. The first antibody of claudin-1 and ZO-1 was incubated with the sections overnight at 4°C. The second fluorescent antibody was incubated with the sections at room temperature for 30 min. After rinsing with PBS and then staining with DAPI, the paraffin sections were observed and photographed under a fluorescence microscope (Olympus BX43, China).

### 2.13. Immunohistochemistry

4 *μ*m paraffin sections were used for immunohistochemistry, and following the elimination of the endogenous peroxidase activity and antigen reparation, the tissues were sealed with blocking buffer. Then, the tissues were incubated with anti-TNF-*α*, anti-IL-6, and anti-IL-1*β* (Proteintech Group, Shanghai, China) overnight at 4°C and subsequently with the secondary antibody. The DAB chromogen was used for incubation, and then hematoxylin was used for counterstaining. After dehydration of gradient ethanol and sealing slices with neutral gum, slices were observed under a microscope. The positive results were stained in yellow. The images were analyzed using Image-Pro Plus software.

### 2.14. Statistical Method

All the data were statistically analyzed by SPSS 22 software, and the data were expressed as mean ± SD. The comparison between groups was performed by single-factor analysis of variance (ANOVA).

## 3. Results

### 3.1. Quality Control of DOFP

**Figure 1:**
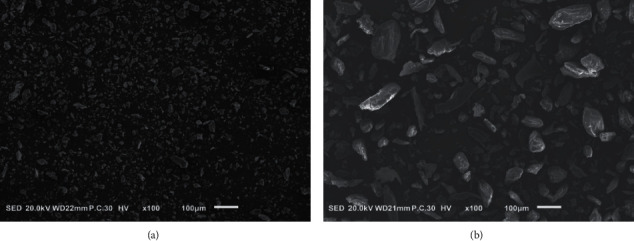
Characteristics of DOFC and DOFP. (a) SEM micrographs of DOFP. (b) SEM micrographs of DOFC.

The DOFP and DOFC were observed and photographed under a scanning electron microscope. There were many intact cells in the DOFC (Figure 1(b)), while DOFP (Figure 1(a)) had fewer intact cells, and most of them were cell fragments. DOFC and DOFP contained 46.50% and 56.67% polysaccharides, respectively, as determined by the phenol sulfuric acid method, which indicated that there was greater polysaccharide dissolution of DOFP as compared to DOFC.

### 3.2. DOFP Relieved the Signs and Symptoms of UC in Rats Induced by DSS

**Figure 2:**
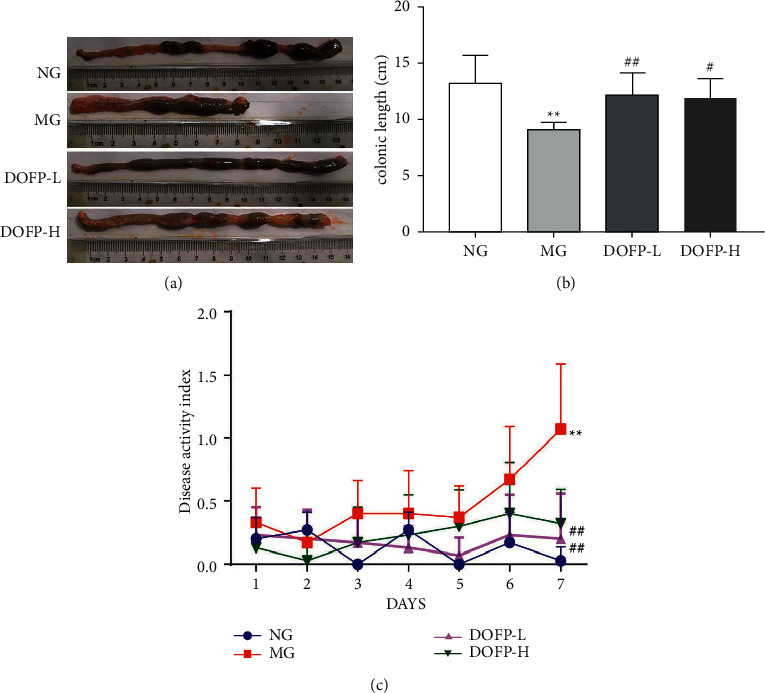
DOFP treatment improves DSS-induced colitis in rats. (a) Representative photos of colon length. (b) Colon length. (c) Disease activity score. ^*∗∗*^*P* < 0.01, compared with the normal group; ^#^*P* < 0.05 and ^##^*P* < 0.01, compared with the model group.

The rats in each group showed different degrees of colitis via symptoms such as weight loss, diarrhea, and hematochezia. Compared with the normal group, the length of colon in the model group was shorter (Figures 2(a) and 2(b)), and the disease activity index (DAI) scores were significantly increased (*P* < 0.05) (Figure 2(c)), suggesting that the model of colitis induced by DSS was successful. Compared with the model group, both high and low doses of DOFP improved the symptoms of colon shortening in model rats, and it significantly improved the DAI score in model rats (*P* < 0.05). The above data indicated that DOFP treatment improved DSS-induced colitis in rats.

### 3.3. The Effect of DOFP on Routine Blood Indexes of DSS-Induced Colitis Model Rats

The results of the blood routine examination of rats showed that WBC, HGB, and PLT in the model group were significantly higher than those in the normal group (*P* < 0.05), and MCV of the model group was significantly lower than that in the normal group (Table 3). Compared with the model group, the levels of WBC, HGB, and PLT in the DOFP-H group were significantly decreased (*P* < 0.05), while the MCV in the DOFP-L and DOFP-H groups was significantly increased (*P* < 0.05). It is suggested that DOFP can reduce WBC and other inflammatory indexes and decrease inflammatory lesions in UC model rats.

### 3.4. The Effect of DOFP on the Pathological Changes in Colon Tissue in DSS-Induced Colitis Model Rats

**Figure 3:**
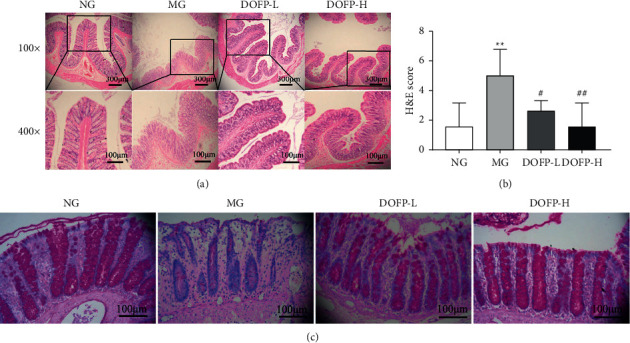
Effects of DOFP on histopathology of colitis rats. (a) Representative picture of colonic H&E staining (100×, 400×). (b) Colonic H&E staining score. (c) Representative picture of colonic PAS staining (400×). ^*∗*^*P* < 0.05 and ^*∗∗*^*P* < 0.01, compared with the normal group; ^#^*P* < 0.05 and ^##^*P* < 0.01, compared with the model group.

The results of H&E staining showed that, compared with the normal group, the colonic mucosa of the model group was severely damaged, a large number of inflammatory cells infiltrated, and the crypt and glandular structures were severely damaged. Compared with the model group, the DOFP-L and DOFP-H groups exhibited decreased colonic mucosal injury, inflammatory cell infiltration, and crypt and glandular structure destruction and other pathological changes of the model rats (Figure 3(a)). The histopathological score of the model group was significantly higher than that of the normal group (*P* < 0.01), and the histopathological score of the DOFP-L and DOFP-H groups was significantly lower than that of the model group (*P* < 0.05, 0.01) (Figure 3(b)).

### 3.5. The Effect of DOFP on Goblet Cells and Mucus in the Colons of DSS-Induced Colitis Model Rats

The results of PAS staining showed that the shape of colonic goblet cells in the normal group was full and round, mucus secretion was abundant, and the mucosal surface was covered with mixed mucus. Compared with the normal group, the goblet cells in the model group atrophied to varying degrees, and the mucus cover on the mucous membrane was decreased. Compared with the model group, the atrophy of intestinal mucosal epithelial cells and goblet cells in the DOFP-L and DOFP-H groups was reversed by varying degrees, and the mucus secretion increased (Figure 3(c)).

### 3.6. Transmission Electron Microscope Observation

**Figure 4:**
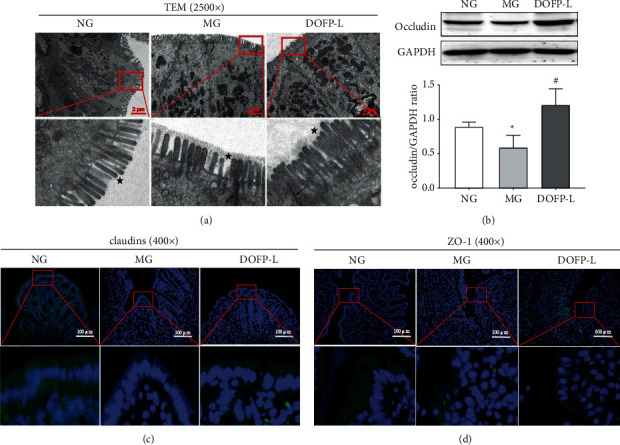
Effects of DOFP on TJ barrier function of colonic mucosa in UC rats. (a) Representative picture of the ultrastructure of colonic mucosa in colon. The arrow points to the tight junction, and the asterisk indicates microvilli. (b) Representative picture and semiquantitative statistical map of occludin protein bands in colon. (c) Representative picture of claudin-1 proteins expression with immunofluorescence in colon (400×). (d) Representative picture of ZO-1 proteins expression with immunofluorescence in colon (400×). ^*∗*^*P* < 0.05 and ^*∗∗*^*P* < 0.01, compared with the normal group; ^#^*P* < 0.05 and ^##^*P* < 0.01, compared with the model group.

The results of TEM showed that the microvilli on the surface of the colonic mucosa epithelial cells in the normal group were intact, the epithelial cells were tightly connected, and the intercellular space exhibited no obvious abnormal changes. In the model group, the rat colonic mucosal epithelial cells lost microvilli, the intercellular space widened, the endoplasmic reticulum expanded, the mitochondria were swollen, part of the cytoplasm was dissolved, and apoptosis was visible. The colonic mucosal epithelial cells in rats from the DOFP group exhibited additional thick and short microvilli that were neatly arranged and uniform in size, the cells were neatly arranged, the degree of intercellular widening was significantly reduced, the glandular epithelia were closely connected to each other, and there was no expanded endoplasmic reticulum or swollen mitochondria (Figure 4(a)).

### 3.7. The Effect of DOFP on the Expression of Occludin Protein in Colon Tissue

Tight junction (TJ) protein plays an important role in maintaining a normal intestinal barrier. In order to clarify the effect of DOFP on TJ protein, the expression of occludin protein in the rat colon was detected by Western blot. The results showed that, compared with the normal group, the model group exhibited significantly decreased expression of occludin in colon tissue (*P* < 0.05). Compared with the model group, the DOFP-L group exhibited significantly increased expression of occludin protein in colon tissue (*P* < 0.05) (Figure 4(b)).

### 3.8. The Effect of DOFP on the Expression of Claudin-1 and ZO-1 Protein in Colon Tissue

The results of immunofluorescence showed that the expression of claudin-1 and ZO-1 protein in the colon tissue of the model group was lower than that of the normal group. Compared with the model group, the expression of claudin-1 and ZO-1 protein in the colon tissue of rats in the DOFP-L group increased (Figures 4(c) and 4(d)). It is suggested that the DOFP can increase the expression of occludin, claudin-1, ZO-1, and other tight junctions in the colon of UC model rats, and this subsequently acts to strengthen the intestinal mucosal barrier.

### 3.9. The Effect of DOFP on the Expression of TNF-*α*, IL-6, and IL-1*β* Protein in Colon Tissue

**Figure 5:**
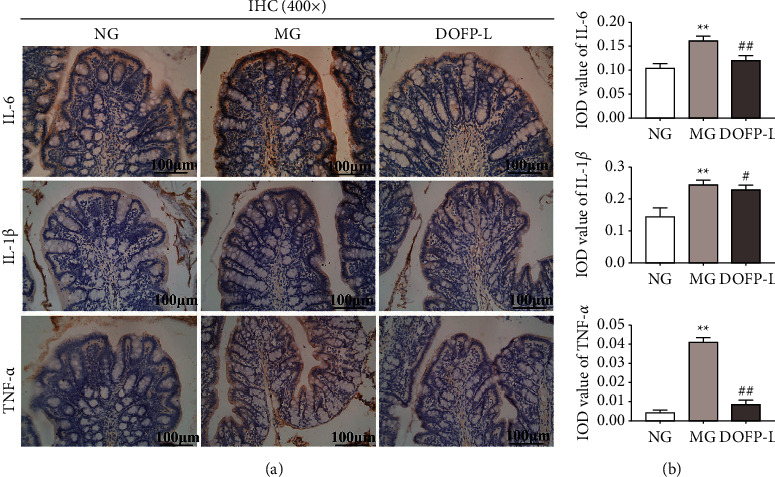
Effects of DOFP on the inflammatory factors TNF-*α*, IL-6, and IL-1*β* of colonic mucosa in UC rats. (a) Representative picture of colonic IHC staining (400×). (b) Colon IHC average optical density statistics chart. ^*∗*^*P* < 0.05 and ^*∗∗*^*P* < 0.01, compared with the normal group; ^#^*P* < 0.05 and ^##^*P* < 0.01, compared with the model group.

The results of immunohistochemistry showed that the expression of TNF-*α*, IL-6, and IL-1*β* protein in the colon tissue of the model group was higher than that of the normal group. Compared with the model group, the expression of TNF-*α*, IL-6, and IL-1*β* protein in the colon tissue of rats in the DOFP-L group decreased (Figures 5(a) and 5(b)). It is suggested that the DOFP can decrease the expression of TNF-*α*, IL-6, and IL-1*β* in the colon of UC model rats and play a role in inhibiting the intestinal inflammatory response.

## 4. Discussion

UC is a chronic inflammatory bowel disease, and its incidence has been increasing worldwide for many years [22]. Increasing evidence shows that the use of Chinese herbal medicine is effective for diseases, especially inflammatory diseases [23]. In the treatment of DSS-induced UC model mice with Lizhong decoction, colonic lesions were decreased in all dose groups, the disease activity index (DAI) score was decreased, and the expression of TJ proteins was upregulated [7]. *Dendrobium officinale* contains a variety of active ingredients, including polysaccharides, alkaloids, glycosides, phenanthrene, bibenzyl, and amino acids, among which polysaccharides have the highest content in *D. officinale*. Our previous literature showed that the total polysaccharide content (47.96%, w/w) and monosaccharide contents (consisted of D-mannose, glucose, galactose, and arabinose at 42.2%, 12.3%, 0.4%, and 0.4%, w/w) were detected in DOFP [18]. The total flavonoid content (3.78 ± 0.48%) and the content of naringenin (0.3601 ± 0.014 mg/g) in DOFP were detected in a sodium hydroxide-aluminum sulfate method and high-performance liquid chromatograph (HPLC) system (1260, Agilent, Germany), respectively, as described in our previous report [19]. Modern pharmacological studies have shown that polysaccharides have anti-inflammatory, antioxidant, antitumor, and other pharmacological effects, enhance immunity, and regulate intestinal flora [24].

*D. officinale* has antifatigue, anti-inflammation, and antioxidation effects, reduces night sweats and dizziness, enhances immunity, and has been used in traditional Chinese medicine for more than 1000 years [23, 25]. *D. officinale* and its polysaccharides have many beneficial anti-UC effects, such as decreasing colonic inflammation and regulating intestinal flora [14, 15].

There are a large number of symbiotic bacteria in the colon. The intestinal mucosal barrier consists of closely arranged colonic epithelial cells that act as an important physical and immune obstacle between the intestinal flora and the body. It is the key factor for the body to maintain the stability of the intestinal environment [26]. Although the exact pathophysiological mechanism of UC is not clear, it has been proposed that the decrease in intestinal mucosal barrier permeability can cause severe inflammation and lead to the development of UC. Therefore, maintaining the integrity of the intestinal mucosal barrier is an important strategy for the treatment of UC [27]. Intestinal mucosal epithelial cells and their TJs form a complete epithelial barrier, which is an important part of the intestinal mucosal mechanical barrier [7].

The barrier function of the intestinal epithelium is partially maintained by the TJs between adjacent epithelial cells. The TJs are composed of a series of proteins with multiple functions, including occludins, claudins, and ZO family proteins. Studies have shown that downregulation of TJs may cause ectopic invasion of intestinal pathogens, antigens, and toxins through the colonic mucosal barrier, resulting in the activation of immune cells and abnormal immune response of the intestinal mucosa. This can destroy the mucosal barrier function and increase intestinal permeability, which can then promote the occurrence and development of UC [28].

Occludins protein is a direct component of the TJ protein structure, and because of its adhesion characteristics, it can form an intercellular barrier through multimerization. For example, with increasing levels of occludins protein, the intercellular adhesion increases, the transmembrane resistance increases, and the permeability of the intestinal mucosal barrier decreases [29]. Both claudins and occludins are cell membrane proteins that are the functional and structural basis of the TJ complex.

Small molecular transmembrane proteins with a relative molecular weight of approximately 22,000 can directly affect the transmembrane resistance of epithelial and endothelial tissues, determine the ion selectivity of the TJ complex, and regulate the TJ permeability [30]. ZO proteins are cytoplasmic proteins that contain PDZ domains, which contribute to the anchoring of TJ proteins such as claudins and occludins to the cytoplasm [31]. Therefore, occludins, claudins, ZO, and other family proteins are often used as indicators to evaluate TJ barrier function and permeability function in tissues and are also important indicators for monitoring the degree of intestinal mucosal barrier injury in UC.

In the current study, it was found that the expression of occludin, claudin-1, ZO-1, and other TJ proteins decreased in DSS-induced UC model rats, and the intestinal mucosal barrier was damaged. In a study on the effect of patchouli extract on DSS-induced UC model rats, the expression of occludin, claudin-1, ZO-1, and other TJ proteins decreased in the model group [23]. In the current study, we found that, after the intervention of DOFP, the protein expression of occludin, claudin-1, and ZO-1 in the colon of model rats was significantly increased, and the colonic mucosal barrier did not degrade. Some studies have shown that *D. officinale* polysaccharides can protect the intestinal mucosal barrier, reduce intestinal cell permeability, maintain intestinal balance, restore intestinal villus damage, and effectively improve intestinal barrier function by upregulating the expression of TJ proteins such as occludin, claudin-1, and ZO-1 and reducing apoptosis [14, 32]. The previous study of our group showed that DOFP significantly increased the expression of occludin, claudin-1, and ZO-1 mRNA, tightened the loose arrangement of intestinal villi, protected the intestinal barrier function, and reduced the liver damage caused by lipopolysaccharide (LPS) in convalescent nonalcoholic fatty liver model mice (induced by a high-sugar and high-fat diet) [18]. DOFP repaired the damaged colonic mucosal barrier by upregulating the expression of occludin, claudin-1, and ZO-1TJ proteins.

DSS-induced UC model rats possess a disease pathology that is similar to that of human UC, including weight loss, diarrhea, hematochezia, intestinal mucosal injury, and other symptoms, in which the severity of hematochezia is one of the important indicators to evaluate the degree of mucosal barrier damage [5]. In the current study, the results of hematoxylin-eosin (H&E) staining and periodic Acid-Schiff (PAS) staining showed that the colon of model rats sustained a mucosal injury, with inflammatory cell infiltration, an increase in goblet cells and mucus, the appearance of other histological lesions, and an increase in the DAI score. Some studies have shown that *D. officinale* polysaccharides can significantly reduce the DAI score of mice with UC induced by DSS and effectively ameliorate the hematochezia and weight loss [14, 33].

Mucosal barrier damage and inflammation are important pathological changes in the course of UC. DSS mainly develops intestinal inflammation by increasing intestinal permeability and damaging the intestinal barrier. It mainly affects the tight junction proteins of epithelial cells such as ZO-1 and occludin to damage the intestinal barrier, thereby increasing TNF-*α* and IL-6 expression of inflammatory factors [34].

In this study, we found that DOFP significantly reduced the DAI score of model rats, reduced the occurrence of hematochezia, reduced the shortening of the colon, improved the disappearance of microvilli of colonic mucosal epithelial cells and expansion of the endoplasmic reticulum, normalized the appearance of the mitochondria, upregulated the expression of TJ protein, downregulated the expression of inflammatory factors, effectively alleviated the injury of the intestinal mucosa, and improved the quality of life of the rats.

## 5. Conclusions

In a word, our results show that DOFP can improve DSS-induced colitis in model rats. It increases the expression of TJ proteins such as occludin, claudin-l, and ZO-1 in colon, inhibits the release of inflammatory factors TNF-*α*, IL-6, and IL-1*β*, repairs intestinal mucosal injury, reduces intestinal cell permeability, and maintain intestinal balance, so as to achieve the purpose of treating UC. These results suggest that *D. officinale* may be developed as a potential drug for the treatment of UC. However, *D. officinale* has a variety of active constituents, such as polysaccharides, flavonoids, alkaloids, and essential oils, which may be the possible active principle of this plant. We can further study it in the future.

### 5.1. Statement of Human and Animal Rights

All of the experimental procedures involving animals were conducted in accordance with the Institutional Animal Care guidelines of Zhejiang University of Technology, China, and approved by the Animal Care and Use Committee of Zhejiang University of Technology, China.

## Figures and Tables

**Table 1:** Scoring standard of the disease activity index (DAI) [20].

Score	Body weight decrease rate (%)	Fecal property	Hematochezia status
0	0	Normal	Normal
1	1–5	Semiloose (+)	Feces with occult blood (+)
2	6–10	Semiloose (++)	Feces with occult blood (++)
3	11–15	Loose (+)	Bloody feces (+)
4	>15	Loose (++)	Bloody feces (++)

**Table 2:** Histological scoring system [21].

Score	Inflammation	Mucosal damage	Crypt damage	Range of lesions (%)
0	None	None	None	0
1	Mild	Mucous layer	1/3	1–25
2	Moderate	Submucosa	2/3	26–50
3	Severe	Muscularis and serosa	100%	51–75
4	—	—	100% + epithelium	76–100

**Table 3:** Effect of DOFP on blood indexes of rats.

Project	NG	MG	DOFP-L	DOFP-H
WBC (10^9^/L)	8.95 ± 1.31	12.35 ± 3.49^*∗*^	10.39 ± 1.14	9.87 ± 1.82^#^
RBC (10^12^/L)	10.26 ± 0.52	10.80 ± 0.90	10.65 ± 0.60	10.13 ± 0.80
HGB (g/L)	185.11 ± 6.25	193.89 ± 11.49^*∗*^	194.70 ± 9.93	181.00 ± 11.80^#^
HCT (%)	54.38 ± 2.70	56.70 ± 3.23	56.57 ± 2.90	54.41 ± 3.30
MCV (Fl)	54.63 ± 2.92	51.91 ± 2.00^*∗*^	53.80 ± 1.55^#^	54.32 ± 2.17^#^
MCH (pg)	18.23 ± 0.87	17.99 ± 0.65	18.29 ± 0.64	18.25 ± 0.59
MCHC (g/L)	343.80 ± 6.66	342.00 ± 4.18	344.20 ± 5.25	339.00 ± 8.25
PLT (10^9^/L)	853.00 ± 153.72	1011.57 ± 123.19^*∗*^	961.88 ± 179.04	837.86 ± 214.65^#^
MPV (fL)	7.61 ± 0.31	7.68 ± 0.24	7.44 ± 0.34^#^	7.55 ± 0.42
EO (%)	0.10 ± 0.02	0.10 ± 0.04	0.11 ± 0.05	0.12 ± 0.04

^*∗*^ *P* < 0.05 and ^*∗∗*^*P* < 0.01, compared with the normal group; ^#^*P* < 0.05 and ^##^*P* < 0.01, compared with the model group.

## Data Availability

The data to support the findings of this study are included within the article. Other data used to support the findings of this study are available from the corresponding author upon request.
